# Optimization of AlCrSiWN Coating Process Parameters and Performance Study by the Matrix Analysis Method

**DOI:** 10.3390/ma15155153

**Published:** 2022-07-25

**Authors:** Shasha Wei, Renxin Wang, Hu Yang, Ziming Guo, Rongchuan Lin, Qingmin Huang, Yuhui Zhou

**Affiliations:** College of Marine Equipment and Mechanical Engineering, Jimei University, Xiamen 361021, China; 200461000025@jmu.edu.cn (S.W.); wrx15828950828@163.com (R.W.); yh980609@163.com (H.Y.); 15238021625@139.com (Z.G.)

**Keywords:** arc ion plating, AlCrSiWN coating, orthogonal experiment, matrix analysis method, coating performance

## Abstract

An AlCrSiWN coating was prepared on a cemented carbide substrate by the arc ion plating technology. The optimization of the coating process was carried out by matrix analysis of orthogonal experiments to calculate the influence of the process parameters on the hardness, bonding and roughness indexes of the coating, determine the optimal coating process parameters, and focus on the influence of the bias voltage on the microscopic morphology, mechanical properties and friction properties of the coating. The results showed that the influence of the process parameters on the indexes of the orthogonal experiments was in the following order: bias voltage > arc current > N_2_ flow rate. The optimal solution was achieved with an arc current of 160 A, a bias voltage of −80 V, and a N_2_ flow rate of 600 sccm. Properly increasing the bias voltage improved the microscopic morphology, mechanical properties and wear resistance of the coating. When the bias voltage was −80 V, the coating surface presented fewer large particles with a less uniform size and no obvious crater defects; in addition, the cross-sectional structure changed from grape-like to columnar, and the coating had higher hardness, lower roughness and better bond strength. In the friction performance test, coating at a −80 V bias voltage showed better wear resistance, which was reflected in lower friction coefficient and wear, and the wear mechanism mainly consisted of adhesion and oxidation wear.

## 1. Introduction

In recent years, carbide composite tools have been highly sought after by the industry for their excellent cutting performance and widely used for machining titanium alloys [1], bearing steel [2] and other materials [3,4]. In contrast, carbide tools suffer from severe tool wear, short service life and poor machining quality when used for difficult-to-cut materials such as printed circuit boards and ceramic-based composites [5]. Many studies have found that depositing a coating on the surface of carbide tools provides the tool with excellent overall performance [6] and service life [7], and the use of coated tools can substantially improve machining quality [8]. Li et al. [9] deposited TiN coatings on carbide tools to improve their cutting performance. Liu et al. [10] found that depositing a ZrN coating on carbide tools’ surface can extend their durability, while also improving their cutting quality. Although a single-layer coating can improve the cutting performance of carbide tools, there are disadvantages such as poor bonding, lower hardness and rougher surface. Some scholars found that composite-coated carbide tools have a better machining quality compared to single-coated carbide tools [11,12]. Multi-layer composite coatings with high hardness, high corrosion resistance and high wear resistance are widely used and have gradually attracted researchers’ attention. Jing et al. [13] investigated the performance of AlTiN-based composite coatings in dry end milling of hardened steel and found that AlTiN-based composite coatings performed well in reducing cutting forces, preventing adhesive wear and isolating cutting heat and were suitable for dry milling of hardened SKD11. It is possible to reduce surface roughness and improve surface quality using coatings by optimizing the coating film thickness [14] and cutting parameters [15]. The coating performance is influenced by process parameters such as bias voltage [16], arc current [17], substrate temperature [18] and gas flow rate [19] in the preparation of multi-layer composite coatings by arc ion plating process. Therefore, optimization of the coating process parameters is essential to obtain excellent coating performance. When conducting multi-factor experiments to explore the optimal process parameters [20], process optimization is usually carried out using the orthogonal experimental method [21], which can extract key information from a small number of experiments, has high efficiency and is simple and easy to implement [22]. The matrix analysis method is used to calculate and analyze the results to obtain the degree of influence of each factor on the results, and the best solution is achieved by controlling the main influencing factors.

The influence of the process parameters on the performance of AlCrSiWN coatings has not been discussed in the literature. In this study, an AlCrSiWN coating was prepared by the arc ion plating technology for carbide milling cutters for printed circuit board processing, and orthogonal experimental matrix analysis was used to optimize the AlCrSiWN coating properties; the effect of bias voltage on the microscopic morphology, mechanical properties and tribological properties of the coating was examined to finally obtain the best coating process parameters.

## 2. Matrix Analysis Method

Orthogonal experiments investigate three indexes, i.e., coating hardness, film-base bonding force and surface roughness, and the common analysis methods for orthogonal experiments are mainly extreme difference analysis and analysis of variance, which have some limitations for the analysis of multiple indexes of orthogonal experiments. In this paper, we used the matrix analysis method to construct three levels of data according to the orthogonal experiment results, namely, index data, factors data, and levels data. A matrix was constructed to calculate the numerical values of the weights of the three factors and their levels to analyze the orthogonal experimental results and determine the optimal solution of the orthogonal experiment and the degree of influence of the three factors on the coating performance index.

The index layer matrix, factor layer matrix, level layer matrix and experimental index weight matrix had to be defined before data processing, according to the following specific process.

Indicator level matrix: If there are *m* factors in an orthogonal experiment, each factor has *n* levels, and the mean of the experimental indicators at the *j*th level of factor A*_i_* is *k_ij_*.

To determine the trend of the examined indicators, the value of *K_ij_* was obtained according to Equation (1)

(1) $$\begin{array}{c} K_{ij}=\left\{\begin{array}{c} k_{ij}\;A_{i}\in I_{1}\\ \frac{1}{k_{ij}}\;A_{i}\in I_{2} \end{array}\right. \end{array},$$

where *I*_1_ = {the larger the inspection index is, the better}, *I*_2_ = {the smaller the inspection index is, the better}, *i* = {1, 2, …, m}, *j* = {1, 2, …, n}.

Establish the matrix of Equation (2) [23]:

(2) $$M=\left[\begin{array}{ccccc} K_{11} & 0 & 0 & \cdots & 0\\ K_{12} & 0 & 0 & \cdots & 0\\ \vdots & \vdots & \vdots & \ddots & \vdots \\ K_{1n} & 0 & 0 & \cdots & 0\\ 0 & K_{21} & 0 & \cdots & 0\\ 0 & K_{22} & 0 & \cdots & 0\\ \vdots & \vdots & \vdots & \ddots & \vdots \\ 0 & K_{2n} & 0 & \cdots & 0\\ \vdots & \vdots & \vdots & \ddots & \vdots \\ 0 & 0 & 0 & \cdots & K_{m1}\\ 0 & 0 & 0 & \cdots & K_{m2}\\ \vdots & \vdots & \vdots & \ddots & \vdots \\ 0 & 0 & 0 & \cdots & K_{mn} \end{array}\right],$$

Factor layer matrix: Let $T_{i}=\frac{1}{\sum_{j=1}^{n}K_{ij}}$ and build the matrix of Equation (3) [24]:

(3) $$T=\left[\begin{array}{cccc} T_{1} & 0 & \cdots & 0\\ 0 & T_{2} & \cdots & 0\\ \vdots & \vdots & \ddots & \vdots \\ 0 & 0 & \cdots & T_{m} \end{array}\right],$$

Horizontal level matrix: the extreme difference of factor A*_i_* in the orthogonal experiment being *R_i_*, let $\;R_{i}=\frac{r_{i}}{\sum_{i=1}^{m}r_{i}}$ and establish the matrix of Equation (4) [25]:

(4) $$R=\left[\begin{array}{c} R_{1}\\ R_{2}\\ \vdots \\ R_{m} \end{array}\right],$$

Experimental indicator weight matrix [26]:

(5) $$W=\mathrm{MTR}=\left[\begin{array}{c} W_{1}\\ W_{2}\\ \vdots \\ W_{m} \end{array}\right],$$

where *W_i_* is the ratio of the index value of the *i*th level of factor A*_i_* to the sum of the index values of all levels of each factor. The calculation can provide the weight values of different levels of each factor, determine the degree of influence of different levels of each factor on the indexes of the orthogonal experiment, and finally allow the selection of the optimal solution.

## 3. Materials and Methods

### 3.1. Coating Preparation Process

Carbide was chosen as the base material for the experiment, and its chemical composition is shown in Table 1. Firstly, the substrate material was polished by using 120, 320, 600, 1000, 1500 and 2000 mesh water-grit sandpaper, and when the visible scratches produced in the previous step were completely removed, the sandpaper was replaced with a higher-grit sandpaper until a mirror finish was achieved. We used a 2CRD200 equipment (Novatec, Padova, ltaly) for cleaning, employing in turn, a cleaning solution and deionized water for slots 1 to 4, i.e., the cleaning solution with alkaline cleaning agents 1153 and 1510 in No. 1 slot and with alkaline cleaning agents 1153 and 1401 in No. 2 slot, and deionized water in No. 3 and No. 4 slots, cleaning the whole process for a total of 54 min. The processed specimens were clamped on the grate and then subjected to coating deposition (deposition equipment from ICS, Milan, ltaly). The whole coating process was divided into four parts:

(1) Furnace cavity temperature rise: before coating deposition, the vacuum of the equipment cavity was fixed at 0.5 Pa, and then the temperature gradient of the furnace cavity was increased by controlling the heating tube; the gradient temperature was set to 350 °C and 420 °C for 10 min and 60 min of insulation, respectively.
(2) Gas cleaning: we waited for the completion of insulation, passed 200 sccm of argon gas, set the bias voltage to −700 V and cleaned the target and furnace cavity for 4 min.
(3) Ion etching: we waited for the completion of cleaning, raised the bias voltage to −800 V, maintained the vacuum level at 0.5 Pa, the argon flow rate at 200 sccm, and the temperature at 420 °C, passed a 90 A current to the Cr target and carried out ion etching on the workpiece for a total of 12 min.
(4) Coating deposition: after ion etching was completed, the argon flow was turned off, and nitrogen was introduced into the furnace chamber to provide N elements. Cr target, AlCrSiW target and AlCr target are used in the deposition process. AlCrSiWN coatings with different process parameters were prepared by varying arc current, bias voltage and N_2_ flow rate.

### 3.2. Orthogonal Experimental Design

We selected the three parameters of arc current, bias voltage and N_2_ flow rate as the influencing factors of the orthogonal experiment and three values for each of them. An L9 (3^4^) orthogonal table was chosen for the experiment, and the experimental scheme was obtained by arranging and combining the influencing factors according to the orthogonal Table 2. The used the letters A, B and C to represent arc current, bias voltage and N_2_ flow rate, respectively.

### 3.3. Structural and Performance Characterization Methods

The surface morphology and cross-sectional morphology of the coatings were observed by scanning electron microscopy (SEM, Crossbeam 550, ZEISS, Jena, Germany) at 10 μm and 4 μm scales, respectively. A laser confocal microscope (VK-X1000, KEYENCE, Ōsaka, Japan) was used to observe the three-dimensional micromorphology of the substrate and coating at a magnification of 400x and to obtain the surface roughness value Ra. The coating hardness was determined using a Vickers hardness tester (FALCON 500, INNOVATEST, Shanghai, China) with a set load of 25 gf, a loading time of 10 s, a test temperature of room temperature, and a quadrilateral cone diamond indenter (126°) at five randomly selected points on the surface of the carbide coated specimen; the average value was taken as the coating hardness. The bond strength of the coating was characterized by its indentation using a Rockwell hardness tester and a diamond indenter with a taper angle of 120°, applying a load of 150 kgf for 5 s. The indentation characteristics were observed using a microscope, and the bond strength level was determined using a standard comparison table. The bond strength of the coating was determined using a coating adhesion auto-scratcher (WS-2005, ZKKH, Lanzhou, China) with a Rockwell C-type diamond stylus (120° and 0.2 mm radius of the spherical tip), a load loading range of 0–150 N, a scratch length of 5 mm, a scratch speed of 5 mm/min and a loading rate of 150 N/min.

The tribological properties of the films were determined by a tribological wear test machine (HT-600, HHIT, Lanzhou, China) with 4 mm-diameter ZrO_2_ balls, a stroke of 10 mm, a motor speed of 300 r/min, and an applied load of 8 N, in a 30 min test. The abrasion profiles were obtained by a laser confocal microscope (VK-X1000, KEYENCE, Ōsaka, Japan), and the wear rate *K* was calculated according to the wear equation:

(6) $$K=\frac{\Delta V}{F\times S},$$

where *K* denotes the volumetric wear rate (mm^3^/N·m), *F* denotes the loading load (N), Δ*V* denotes the volume amount of wear (mm^3^), and *S* denotes the friction length (m). The values of the friction factor and wear rate of the coating were determined. In addition, the wear morphology of the specimens was characterized by scanning electron microscopy (Phenom-XL, RESINA, Shanghai, China), the wear spot morphology of the grinding balls was observed by optical microscopy, and the elemental distribution of the wear surface was analyzed by energy spectroscopy.

## 4. Results and Discussion

### 4.1. Statistics of the Orthogonal Experimental Results and Matrix Analysis

The experiments used a FALCOON 500 Vickers hardness tester to measure the hardness of the coating, a WS-2005 automatic scratch tester to determine the size of the bond between the coating and the substrate, and a VK-X1000 laser confocal microscope to measure the surface roughness of the coating. The resulting values of the three performance indexes are summarized in Table 3.

The ***M***_1_, ***T***_1_ and ***R***_1_ matrices were obtained from the data in Table 3 for microhardness.

$$M_{1}=\left[\begin{array}{ccc} 3380.9 & 0 & 0\\ 3753.4 & 0 & 0\\ 3663.4 & 0 & 0\\ 0 & 3671.4 & 0\\ 0 & 3580.0 & 0\\ 0 & 3546.3 & 0\\ 0 & 0 & 3608.2\\ 0 & 0 & 3571.2\\ 0 & 0 & 3618.2 \end{array}\right]\;\;\;\;\;T_{1}=\left[\begin{array}{ccc} \frac{1}{10797.7} & 0 & 0\\ 0 & \frac{1}{10797.7} & 0\\ 0 & 0 & \frac{1}{10797.7} \end{array}\right]R_{1}=\left[\begin{array}{c} 0.6840\\ 0.2297\\ 0.0863 \end{array}\right]$$

Weight matrix of hardness indicators:

$$W_{1}=M_{1}T_{1}R_{1}=\left[\begin{array}{c} 0.2142\\ 0.2378\\ 0.2321\\ 0.0781\\ 0.0762\\ 0.0755\\ 0.0288\\ 0.0285\\ 0.0289 \end{array}\right]$$

Similarly, the weight matrix of the adhesion index were obtained as follows:

$$W_{2}=M_{2}T_{2}R_{2}=\left[\begin{array}{c} 0.0660\\ 0.0733\\ 0.0652\\ 0.1502\\ 0.1733\\ 0.2037\\ 0.0814\\ 0.0916\\ 0.0953 \end{array}\right]$$

The ***M***_3_, ***T***_3_ and ***R***_3_ matrices were obtained from the data of surface roughness in Table 3.

$$M_{3}=\left[\begin{array}{ccc} 0.0077 & 0 & 0\\ 0.0077 & 0 & 0\\ 0.0077 & 0 & 0\\ 0 & 0.0073 & 0\\ 0 & 0.0078 & 0\\ 0 & 0.0080 & 0\\ 0 & 0 & 0.0079\\ 0 & 0 & 0.0075\\ 0 & 0 & 0.0077 \end{array}\right]\;\;T_{3}=\left[\begin{array}{ccc} 43.466 & 0 & 0\\ 0 & 43.391 & 0\\ 0 & 0 & 43.446 \end{array}\right]\;R_{3}=\left[\begin{array}{c} 0.0464\\ 0.6288\\ 0.3248 \end{array}\right]$$

Then, the weight matrix of the surface roughness index was obtained:

$$W_{3}=M_{3}T_{3}R_{3}=\left[\begin{array}{c} 0.0154\\ 0.0155\\ 0.0154\\ 0.1980\\ 0.2120\\ 0.2188\\ 0.1107\\ 0.1052\\ 0.1089 \end{array}\right]$$

Total weight matrix:

$$W=\frac{W_{1}+W_{2}+W_{3}}{3}=\left[\begin{array}{c} 0.0985\\ 0.1088\\ 0.1042\\ 0.1421\\ 0.1538\\ 0.1660\\ 0.0737\\ 0.0751\\ 0.0777 \end{array}\right]=\left[\begin{array}{c} A_{1}\\ A_{2}\\ A_{3}\\ B_{1}\\ B_{2}\\ B_{3}\\ C_{1}\\ C_{2}\\ C_{3} \end{array}\right]$$

The results obtained from the weight matrices of the three indexes showed that for the three levels of factor A, we found A_1_ = 0.0985, A_2_ = 0.1088 and A_3_ = 0.1042, of which, the weight of A_2_ was the largest; similarly, for factor B, the weight of B_3,_ i.e., 0.1660 was the largest, and for factor C, the weight of C_3_ i.e., 0.0777, was the largest. Therefore, the order of influence was B > A > C, that is, bias voltage > arc current > N_2_ flow.

### 4.2. Surface and Cross-Sectional Morphology

**Figure 1:**
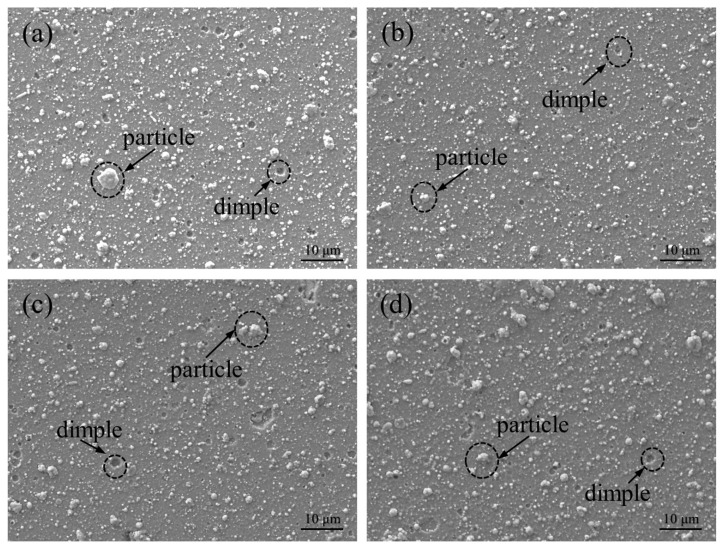
Surface morphology of the coating at different bias voltages: (**a**) −60 V; (**b**) −80 V; (**c**) −100 V; (**d**) −120 V.

**Figure 2:**
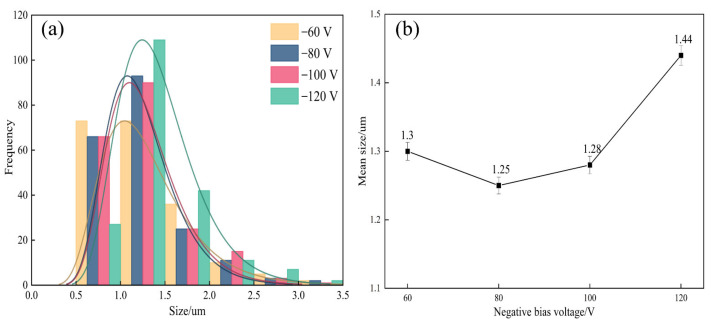
Statistical plots describing the large particles on the coating’s surface under different bias voltages: (**a**) histogram of frequency distribution; (**b**) trend of average particle size.

The surface morphology of the AlCrSiWN coatings with different bias voltages was observed by scanning electron microscopy, as shown in Figure 1. It can be seen in the figure that the surface of the coating prepared under the four sets of bias voltage conditions was smooth and flat, and there were no large bubbles, spalling and obvious defects such as uncoated parts. At the same time, the coating surface also had the typical characteristics obtained by ion-coating, i.e., it presented large particles of different sizes. The size of the large particles was determined by ImageJ software, as shown in Figure 2. It can be seen that the size of the large particles on the coating surface under the four values of bias voltage was mainly distributed from 0.5 μm to 3.5 μm. When the bias voltage was −60 V, the number of large particles on the coating surface was high, and most of them had a size between 0.5 μm and 2.0 μm, with an average size of about 1.30 μm; in addition, the surface crater’s size was small. When the bias voltage was −80 V, the number of large particles on the surface decreased, and their size was mainly distributed in the range of 0.5 μm to 1.5 μm, with an average size of about 1.25 μm, which was 3.8% less than the average size observed at −60 V. When the bias voltage increased to −100 V, the number and size of the large particles increased, and the average size was 1.28 μm. When the bias voltage was −120 V, statistical analysis showed a significant increase in the number of large particles. We found that the number of particles with sizes between 1.5 μm and 2.0 μm was about 160, and their average size increased to 1.44 μm. The analysis suggested that the generation of large particles was caused by atoms or ions aggregated during the deposition process, and their size depended on the degree of collision of atoms or ions emitted by the target material [27]. In the process of depositing coatings by arc ion plating, the substrate is negatively charged compared to the plasma, which thus has a repulsive effect on negatively charged droplets, and when the bias voltage increases, the repulsive effect is enhanced and the number of larger droplets reaching the surface of the substrate decreases. Therefore, when the bias voltage increased to −80 V, the average size of the large particles decreased. When the bias voltage was further increased, the formation of large droplets on the coating surface was intensified; on the other hand, the increase of bias voltage enhanced the ion bombardment, which sputtered the surface droplets, thus producing pits. The above analysis showed that when the bias voltage was −80 V, the number and size of large particles on the surface of the coating was the smallest, there was no obvious crater defect, and the surface was smooth and flat; therefore the surface quality was optimal.

**Figure 3:**
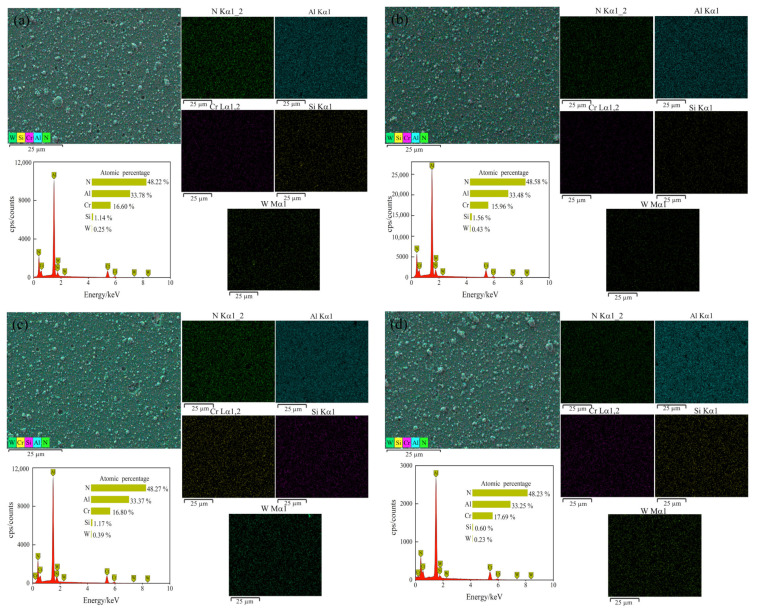
Elemental surface scan profiles for different bias coatings: (**a**) −60 V; (**b**) −80 V; (**c**) −100 V; (**d**) −120 V.

The surface elemental profiles of the coatings at different bias voltages are shown in Figure 3. From the elemental surface distribution, it can be seen that the coating was mainly composed of Al, Cr, Si, W and N. The constituent elements were evenly distributed on the coating surface, with N and Al elements densely distributed, and other elements sparsely distributed on the surface. From the element content of the coating surface under different bias voltage shown in Table 4, it can be seen that the content of the five constituent elements of the coating did not change significantly. We found that N was about 48 at.%, Al was about 34 at.%, Cr was about 17 at.%, Si and W were about 2 at.%. The elemental content of W and Si remained stable, and the atomic ratio of Cr/Al remained around 0.5, larger than the atomic ratio of Cr/Al in the target material, indicating that an obvious segregation phenomenon occurred. Generally speaking, elements with a high melting points tend to show positive segregation, while elements with a low melting points show negative segregation [28]. The melting point of Al is much lower than that of Cr, so Al content in the coating decreased relative to the target material, and Cr content increased.

**Figure 4:**
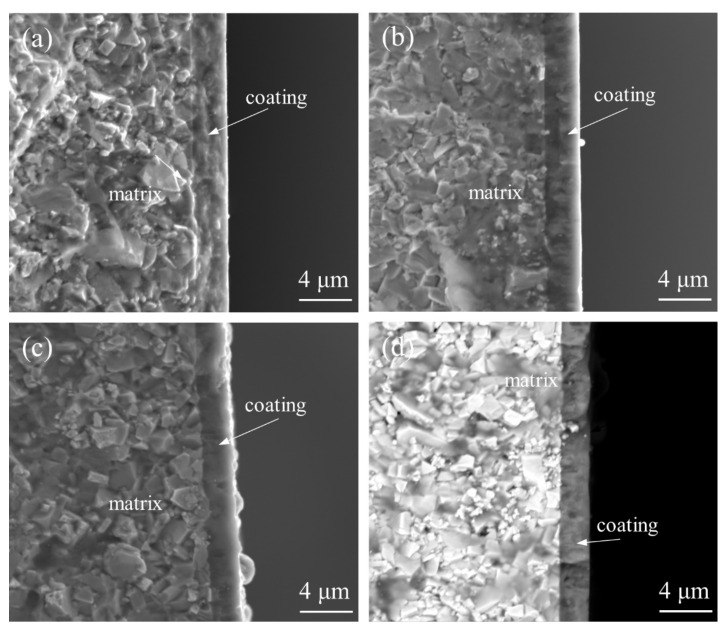
Cross-sectional morphology of the coatings at different bias voltages: (**a**) −60 V; (**b**) −80 V; (**c**) −100 V; (**d**) −120 V.

Figure 4 shows the cross-sectional morphology of the coating under different bias voltages. It can be seen that the coating adhered closely to the carbide substrate under the four bias voltages without obvious delamination, and the bonding strength of the coating was good. The total thickness of the coating was around 2.5 μm at −60 V and −80 V bias voltage and decreased when the bias voltage was increased to 100 V and −120 V, mainly because the bombardment energy of the ions increased with the increase of the bias voltage, the bombardment effect was more intense, and the deposition efficiency decreased. The coating at a bias voltage of −60 V showed a grape-like structure, and the film structure was not dense enough. When the bias voltage was −80 V, the coating was dense and smooth without obvious defects, mainly showing a columnar structure. When the bias voltage increased to −100 V, defects started to appear in the cross section, and when the bias voltage increased further, the defects produced by the ion bombardment increased significantly.

### 4.3. Hardness and Roughness

**Figure 5:**
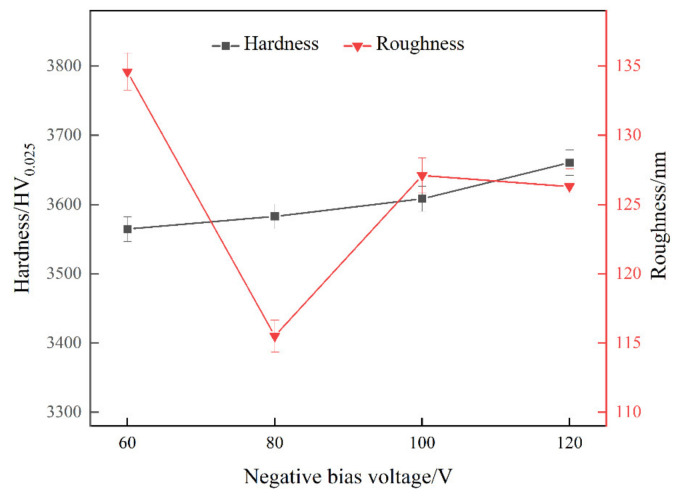
The trends of hardness and roughness of the coatings at different negative bias voltages.

The variation of hardness and roughness of the coatings at different bias voltages are shown in Figure 5. It can be seen that with the increase of bias voltage, the Vickers hardness value of the coating showed a linear increase, and the minimum and maximum values of the coating hardness were obtained at bias voltages of −60 V and −120 V, corresponding to 3564.4 HV_0.025_ and 3660.5 HV_0.025_, respectively, and showing an increase of 2.7%, indicating that the change of bias voltage improved to a certain extent the hardness of the coating. However, too high a bias voltage would result in more defects, preventing a further increase in the hardness. The surface roughness Ra of the coatings at the four bias voltages were 134.6 nm, 115.5 nm, 127.1 nm and 126.3 nm, respectively, and it was found that the lowest roughness of the coatings was obtained when the bias voltage was −80 V.

### 4.4. Bonding Strength

**Figure 6:**
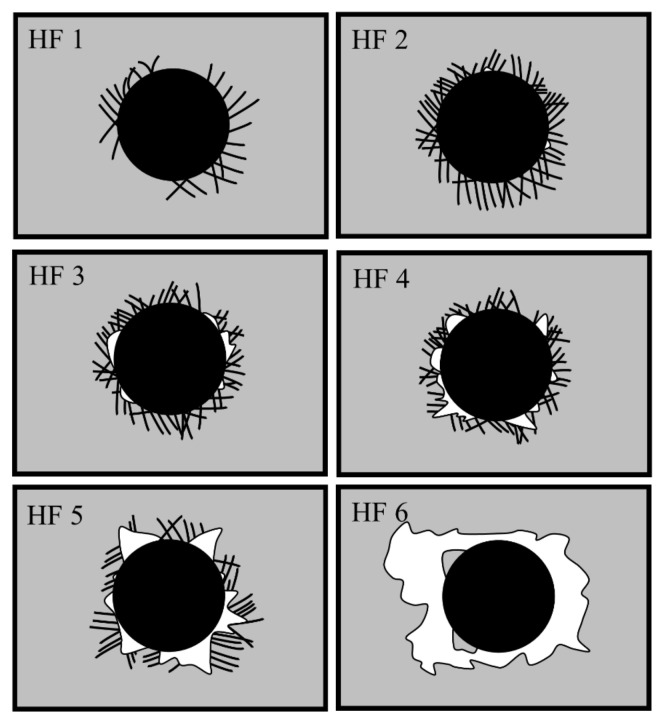
Comparison of the indentation bond strength.

**Figure 7:**
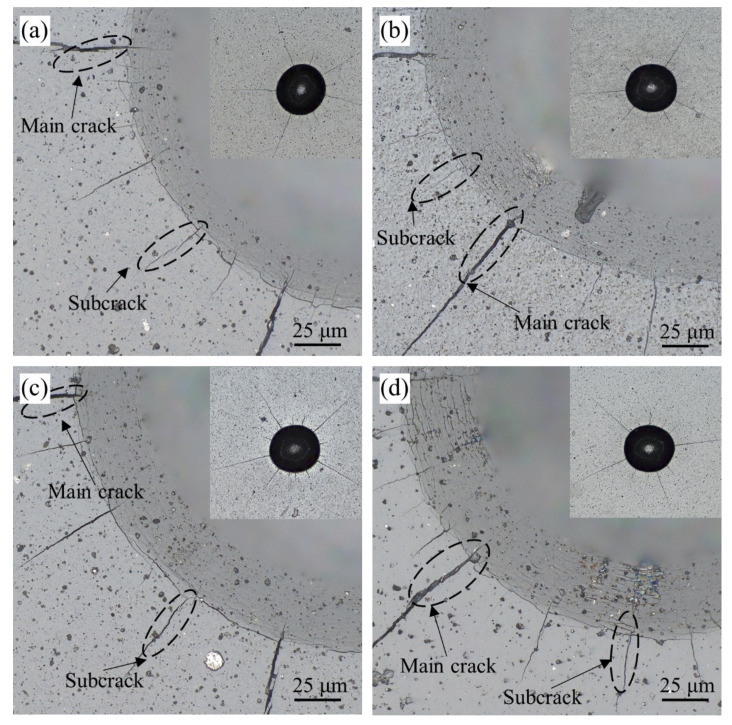
Indentation profiles of the coatings at different bias voltages: (**a**) −60 V; (**b**) −80 V; (**c**) −100 V; (**d**) −120 V.

In the high-speed milling process of PCB, the bonding strength of coating and substrate has an important influence on the life of coated milling cutters. If the bonding strength of coating and substrate is poor, coating delamination and spalling will become the main form of failure, resulting in a shorter service life of the coated tools. According to the determination criteria (Figure 6), it can be seen that the bond strength could be classified into HF1–HF6 levels, in order from high to low. We observed cracks around the indentation (HF1~HF6), coating spalling (HF5, HF6) and other types of damage. When the conical diamond indenter was pressed against the specimen, both the coating and the substrate showed different degrees of plastic deformation. In addition, a significant radial tensile strain occurred in the coating and substrate when applying the load, and circumferential cracks in different numbers and with different lengths appeared at the edges of the indentation area. From the indentation morphology of the coatings under different bias voltages, shown in Figure 7, it was found that the coatings at the four bias voltages did not show spalling or delamination, and circumferential cracks of different lengths and in different numbers were distributed around the indentation. The cracks in all the four coatings formed and expanded radially along the center of the indentation, producing radial cracks around them. On average, we found four main cracks with sub-cracks of shorter lengths. The number of cracks around the indentation and the length of the cracks were reduced when the bias voltage was −80 V compared with other bias voltages, indicating that the coating with an arc current of −80 V had better bonding strength.

### 4.5. Frictional Properties

**Figure 8:**
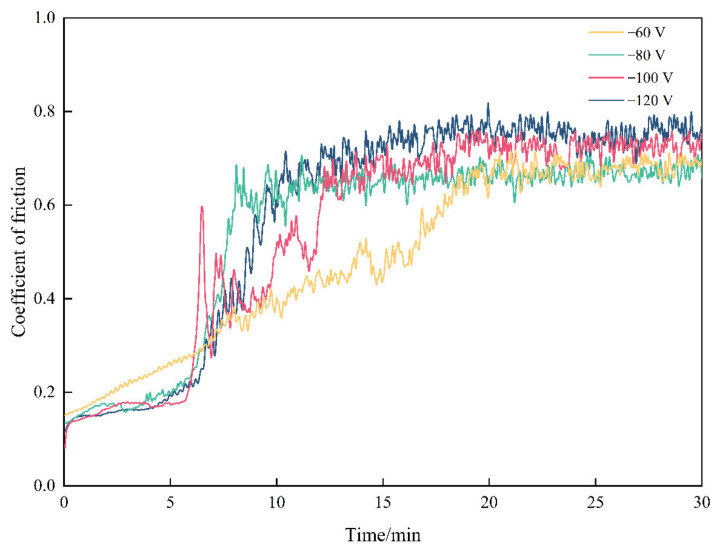
Friction coefficient curve of the coating.

The friction coefficient curves of the coatings t different bias voltages are shown in Figure 8. In the figure, it can be seen that when the bias voltage was −60 V, the friction coefficient of the coating grew slowly within 0~20 min and then fluctuated, stabilizing at about 0.68. When the bias voltage was −80 V, within the first 0~10 min, the coating was in the initial stage of wear, and the friction coefficient increased rapidly, mainly due to the low surface roughness of the coating, few initial contact points between the ball and the disk, and a small actual contact area. The plowing effect of the friction ball and the micro-convex body on the surface of the coating caused the initial friction coefficient to increase rapidly first, and then in 10~30 min, a stable wear stage was reached. Within 30 min in the stable wear stage, the coating surface of the micro-convex body peak gradually wore away, the surface roughness decreased, the contact area increased, while the overall friction coefficient remained relatively smooth, and the fluctuation was not large, stabilized at about 0.66. When the bias voltage increased to −100 V and −120 V, the friction coefficient curve tended to stabilize around 16 min, but the curve fluctuated more, and the friction coefficient varied between 0.65 and 0.75.

**Figure 9:**
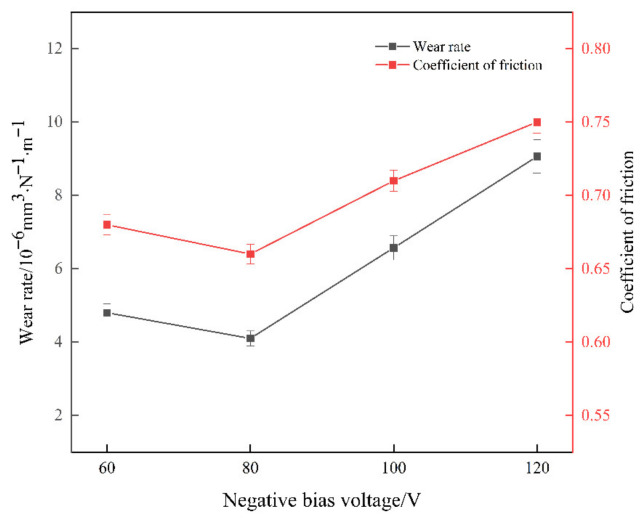
Coating wear rate and average friction coefficient curves at different bias voltages.

Figure 9 shows the average friction coefficient and wear rate of the coating at different bias voltages. The average friction coefficient of the coating showed a trend of decreasing and then increasing with the increase of the bias voltage. The lowest average friction coefficient of the coating was 0.66 at −80 V and reached the maximum value of 0.75 at a bias voltage of −120 V. According to the wear curves, the wear rates of the coating samples at different bias voltages were 4.80 × 10^−6^ mm^3^·N^−1^·m^−1^, 4.10 × 10^−6^ mm^3^·N^−1^·m^−1^, 6.57 × 10^−6^ mm^3^·N^−1^·m^−1^, 9.57 × 10^−6^ mm^3^·N^−1^·m^−1^ and 9.57 × 10^−6^ mm^3^·N^−1^·m^−1^. The lowest wear rate was observed when the bias voltage was 80 V, and the highest wear rate was observed when the bias voltage was −120 V. Both the average friction coefficient and the wear rate showed a consistent trend, i.e., they decreased and then increased. When the bias voltage was −80 V, the friction coefficient of the coating was smaller, and the wear rate was lower, which was mainly due to the better surface quality of the coating; the coating quickly reached a stable wear state during the wear process, which reduced the wear amount.

**Figure 10:**
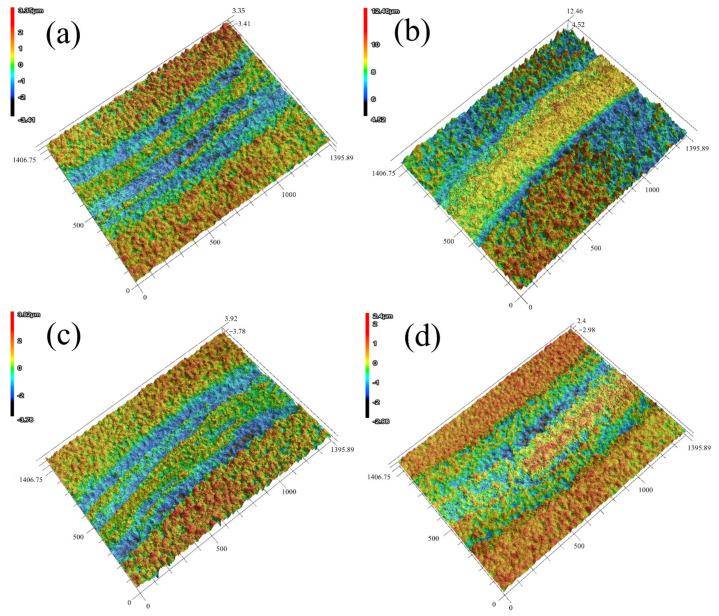
Three-dimensional morphology of abrasion marks of coatings at different bias voltages: (**a**) −60 V; (**b**) −80 V; (**c**) −100 V; (**d**) −120 V.

Figure 10 shows the three-dimensional shape of the abrasion marks obtained from the friction wear test of the coating under different bias voltages. In the figure, it can be seen that when the bias voltage was −60 V, −100 V and −120 V, the coating presented large gully-like abrasion marks, and the frictional adhesion phenomenon could be observed from the trajectory of the abrasion marks. When the bias voltage was −80 V, the abrasion marks were smooth and flat, and their width of the abrasion marks was smaller.

**Figure 11:**
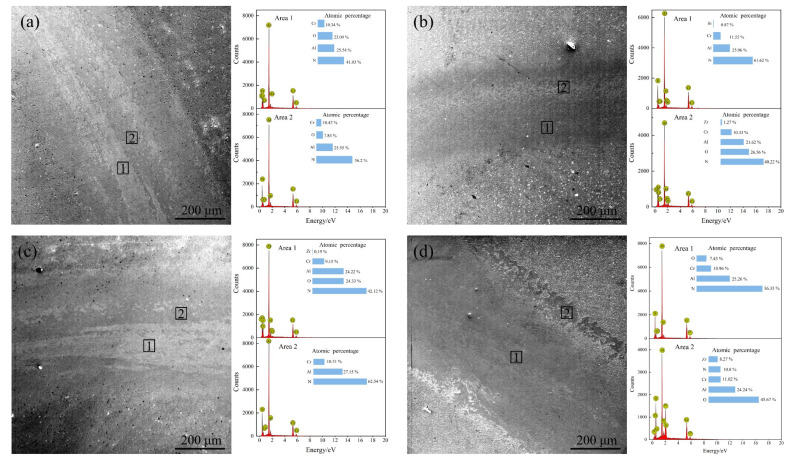
Abrasion morphology of the coatings at different bias voltages: (**a**) −60 V; (**b**) −80 V; (**c**) −100 V; (**d**) −120 V.

The shape of the abrasion marks obtained from the friction wear test of the coating under different bias voltage is shown in Figure 11. In the figure, it can be seen that there were two typical wear patterns, namely, wear track and chip accumulation. The wear track was mainly due to the wear of the coating material, and the chip accumulation was caused by the redeposition of grinding balls and coating debris. When the bias voltage was −60 V, −80 V and −100 V, the generated abrasive chips were distributed in the center of the abrasive marks, and the coating wear mechanism was mainly adhesive wear. At a bias voltage of 60 V, 23.09 at.% of O elements were detected in area 1, implying a severe oxidative wear of the coating, while the central area of the abrasion marks was covered with abrasive chips, showing large adhesion; and the coating wear mechanism was mainly adhesive wear accompanied by local abrasive wear. The frictional abrasion trajectory of the coating at −80 V bias voltage was shallow, the adhesion phenomenon was not obvious, and the width of the final abrasion was small. The wear mechanism was analyzed and appeared to be adhesive wear and oxidative wear. Figure 11c shows the abrasion pattern of the coating at a bias voltage of −100 V. It can be seen that the coating was flat, and the abrasion marks were shallow, in the form of grooves. When the bias voltage was increased to −120 V, the wear marks of the coating became deeper (Figure 11d), the middle area of the wear marks was smooth, abrasive debris accumulated at the boundary, and different levels of O element were detected at the boundary of the wear marks as well as in the middle area of the wear marks, especially at the boundary of the wear marks where the content of O element reached 45.67 at.%. In addition, 8.27 at.% of Zr elements was detected, indicating that the friction process occurred The re-deposition of grinding balls and coating debris at the boundary caused oxide buildup, and severe oxidative wear occurred, i.e., the main friction mechanism of the coating was adhesive wear and oxidative wear.

A comprehensive analysis showed that in the friction process, at the bias voltage of −80 V, adhesive wear and oxidation wear occurred between the coating and the friction ball. The coating was in a stable wear state throughout the friction process, specifically, the coating friction coefficient was stable at about 0.66, and its fluctuation was not large, and the wear rate of the coating was the lowest. The main reason was that the coating had a high hardness, which played a supporting role in the friction process. After the extrusion of the grinding ball, the surface of the film became smoother and denser, while the oxidation film formed during the friction process adhered to the surface of the coating and, after repeated cycles, formed a dense layer on the surface of the coating, which could act as a self-lubricating layer, thus reducing the degree of friction between the coating and the friction ball. Therefore, the AlCrSiWN coating prepared under the bias voltage of −80 V showed better frictional wear performance.

## 5. Conclusions

In this study, an AlCrSiWN coating was prepared on a cemented carbide substrate using the arc ion plating technology, the coating process was optimized by orthogonal experimental matrix analysis, and the effect of the bias voltage on the microscopic morphology, mechanical properties and friction properties of the coating was investigated to obtain a composite coating with excellent performance. Based on the results, the following conclusions were drawn.

(1) The three process parameters affect the experimental indexes in this order of priority: bias voltage > arc current > N_2_ flow rate.
(2) It was found that the surface quality and mechanical properties of the coatings were optimal at a bias voltage of −80 V than at the other bias voltages examined.
(3) After the frictional wear test, it was found that the coating had better frictional properties when the bias voltage was −80 V compared with other bias voltages, and the wear mechanism was mainly adhesive wear and oxidation wear.
(4) We found that the optimum process parameters for the AlCrSiWN coating were as follows: an arc current of 160 A, a bias voltage of −80 V, and a N_2_ flow rate of 600 sccm.

## Figures and Tables

**Table 1:** Cemented carbide specimen composition.

Model	WC%	Co%	Graininess
K05	93.00	6.20	0.4

**Table 2:** Orthogonal experimental protocol.

Programs	Factors		
	A (Arc Current/A)	B (Bias Voltage/V)	C (N_2_ Flow/sccm)
1	200	−120	800
2	200	−100	700
3	200	−80	600
4	160	−120	700
5	160	−100	600
6	160	−80	800
7	120	−120	600
8	120	−100	800
9	120	−80	700

**Table 3:** Results of the orthogonal experiments.

Programs	Factors			Hardness (HV_0.025_)	Adhesion (N)	Roughness (nm)
	A (A)	B (V)	C (sccm)			
1	200	−120	800	3392.2	67.8	140.0
2	200	−100	700	3407.7	81.1	138.7
3	200	−80	600	3342.8	106.1	113.0
4	160	−120	700	3793.4	85.1	130.0
5	160	−100	600	3683.2	102.3	132.3
6	160	−80	800	3783.5	95.7	127.2
7	120	−120	600	3828.6	72.2	143.4
8	120	−100	800	3649.0	76.3	115.1
9	120	−80	700	3521.6	103.5	133.9

**Table 4:** Element content of the coating surface with different bias voltages.

Bias Voltage/V	N/at.%	Al/at.%	Cr/at.%	Si/at.%	W/at.%
−60	48.22	33.78	16.6	1.14	0.26
−80	48.58	33.47	15.96	1.56	0.43
−100	48.27	33.37	16.8	1.17	0.39
−120	48.23	33.25	17.69	0.6	0.23

## Abbreviations

A	Arc current/A
B	Bias voltage/V
C	N_2_ flow rate/sccm
SEM	Scanning Electron Microscopy
EDS	Energy-Dispersive Spectrometry
Ra	Roughness parameters
